# Deviation Analyses of Computer-Assisted, Template-Guided Mandibular Reconstruction With Combined Osteotomy and Reconstruction Pre-Shaped Plate Position Technology: A Comparative Study

**DOI:** 10.3389/fonc.2021.719466

**Published:** 2021-10-27

**Authors:** Jie Chen, Ruipu Zhang, Ye Liang, Yujie Ma, Saiwen Song, Canhua Jiang

**Affiliations:** ^1^ Department of Oral and Maxillofacial Surgery, Center of Stomatology, Xiangya Hospital, Central South University, Changsha, China; ^2^ Research Center of Oral and Maxillofacial Tumor, Xiangya Hospital, Central South University, Changsha, China; ^3^ Institute of Oral Cancer and Precancerous Lesions, Central South University, Changsha, China

**Keywords:** mandibular reconstruction, virtual surgical planning, template-guided surgery, 3D printing, deviation analyses

## Abstract

**Background:**

Computer-assisted and template-guided mandibular reconstruction provides higher accuracy and less variation than conventional freehand surgeries. The combined osteotomy and reconstruction pre-shaped plate position (CORPPP) technique is a reliable choice for mandibular reconstruction. This study aimed to evaluate the accuracy of CORPPP-guided fibular flap mandibular reconstruction and analyze the possible causes of the deviations.

**Patients and Methods:**

From June 2015 to December 2016, 28 patients underwent fibular flap mandibular reconstruction. Virtual planning and personalized CORPPP-guided templates were applied in 15 patients while 13 patients received conventional freehand surgeries. Deviations during mandibulectomy and fibular osteotomy, and overall and triaxial deviation of the corresponding mandibular anatomical landmarks were measured by superimposing the pre- and postoperative virtual models.

**Results:**

The deviation of the resection line and resection angle was 1.23 ± 0.98 mm and 4.11° ± 2.60°. The actual length of fibula segments was longer than the designed length in 7 cases (mean: 0.35 ± 0.32 mm) and shorter in 22 cases (mean: 1.53 ± 1.19 mm). In patients without ramus reconstruction, deviations of the ipsilateral condylar head point (Co.), gonion point (Go.), and coracoid process point (Cor.) were 6.71 ± 3.42 mm, 5.38 ± 1.71 mm, and 11.05 ± 3.24 mm in the freehand group and 1.73 ± 1.13 mm, 1.86 ± 0.96 mm, and 2.54 ± 0.50 mm in the CORPPP group, respectively, with significant statistical differences (*p* < 0.05). In patients with ramus reconstruction, deviations of ipsilateral Co. and Go. were 9.79 ± 4.74 mm *vs*. 3.57 ± 1.62 mm (*p* < 0.05), and 15.17 ± 6.53 mm *vs*. 4.36 ± 1.68 mm (*p* < 0.05) in the freehand group and CORPPP group, respectively.

**Conclusion:**

Mandibular reconstructions employing virtual planning and personalized CORPPP-guided templates show significantly higher predictability, convenience, and accuracy of mandibular reconstruction compared with conventional freehand surgeries. However, more clinical cases were required for further dimensional deviation analysis. The application and exploration of clinical practice would also continuously improve the design of templates.

## 1 Introduction

Mandibular defects can be caused by radical surgery of oral and maxillofacial tumors, osteomyelitis, or trauma of the jaw, and can lead to severe functional and aesthetic deficits, negatively affecting quality of life. Vascularized autologous bone grafting, especially the fibular free flap, serves as the workhorse for segmental mandibular defect reconstruction (1–4). Successful reconstruction includes restoration of symmetrical appearance, sufficient chewing space, and correct joint position, signifying its great demand for accurate position of both the fibula and the remaining mandible (5, 6). However, due to the interruption of the mandibular continuity, even a small displacement may cause the entire jaw to be deflected.

With the aid of computer-assisted surgery (CAS) and three-dimensional (3D) printing technology, template-guided surgeries are gaining increasing popularity (7). Personalized guiding templates can be fabricated, and the titanium plates can be pre-shaped in advance to shorten the operation duration. Template-guided mandibular reconstruction provides higher accuracy, acceptability, and less variation than the conventional free-hand surgeries (8–12).

The combined osteotomy and reconstruction pre-shaped plate position (CORPPP) technique, as previously described, is a reliable choice for mandibular reconstruction (13). In this study, we evaluated the accuracy of CORPPP-guided mandibular reconstruction with fibula free flaps and analyzed the possible causes of the deviations. Furthermore, we applied coordinate conversion to analyze the deviations between the designed model and the actual model in triaxial directions. Results were analyzed to provide suggestions for further accuracy improvement of mandibular reconstruction.

## 2 Patients and Methods

### 2.1 Clinical Characteristics of Patients

This study was approved by the Center of Medical Ethics of Central South University (Changsha, China; serial number 201512515). Written informed consents were obtained from the patients in accordance with the Declaration of Helsinki. All methods were performed in accordance with the relevant guidelines and regulations approved by the institutional review board of the Center of Medical Ethics of Central South University.

From June 2015 to December 2016, 28 patients were recruited in this study and underwent segmental mandibulectomy. Clinical characteristics of all the patients are presented in **Table 1**. Reconstruction with fibula free flaps were conducted either by the conventional freehand procedure (freehand group, 15 patients) or the CORPPP-guided surgical templates (CORPPP group, 13 patients) based on their personal preference. The mandible defects after surgical resection were classified by the Urken’s CRBS (Condyle, Ramus, Body, Symphysis) classification criteria (14).

**Table 1:** Clinical characteristics of the patients.

	Freehand group	CORPPP group
**Number of cases**	15	13
**Gender**		
Male	10	8
Female	5	5
**Age**		
Range	29–67	21–64
Mean ± SD	43.4 ± 13.1	38.9 ± 14.4
**Mandibular lesions**	Ameloblastoma/9	Ameloblastoma/9
	Ossifying fibroma/1	Gingival cancer/1
	Oral malignancies/5	Osteoradionecrosis/1
		Other benign tumors/2
**Whether the ramus was reconstructed**		
Yes	8	7
No	7	6
**Number of cases by Urken’s classification**		
BR	7	6
S^H^BR	1	1
B	2	1
S^H^B	4	3
BS	1	1
BSB	0	1
**Number of fibula segments**		
1	2	1
2	12	9
3	1	2
4	0	1

### 2.2 Surgical Simulation and Design of the CORPPP Guiding Template

**Figure 1:**
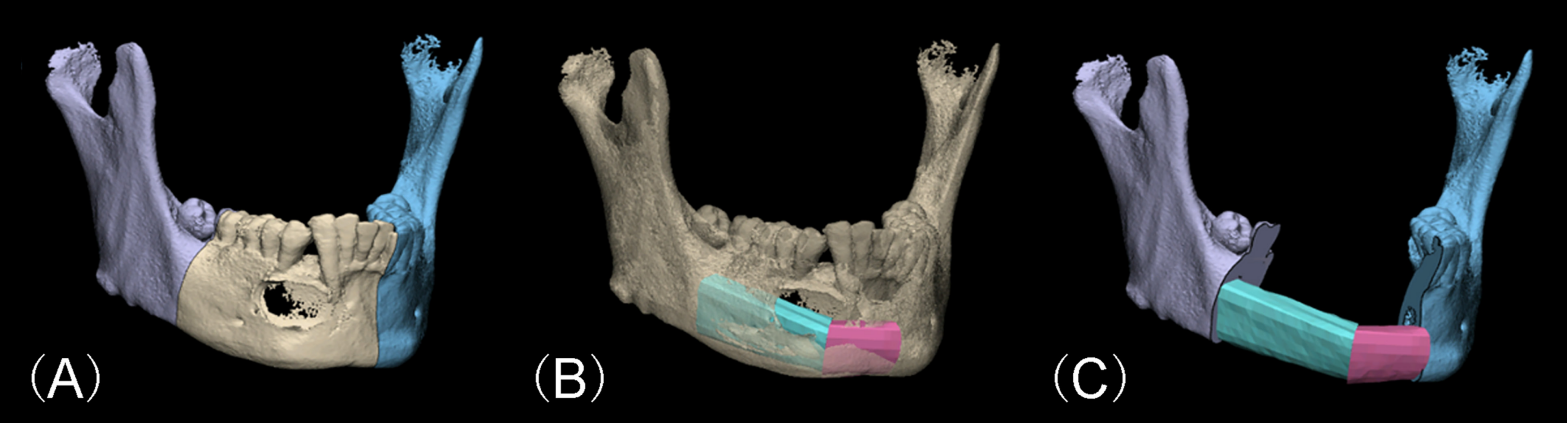
Virtual surgery simulation of mandibular lesion resection and fibular reconstruction. **(A)** Determination of the range of the mandibulectomy. **(B)** Arrangement of the fibula segments into the defect region according to the contour of the original mandible. **(C)** Expected virtual mandibular model after fibular reconstruction.

**Figure 2:**
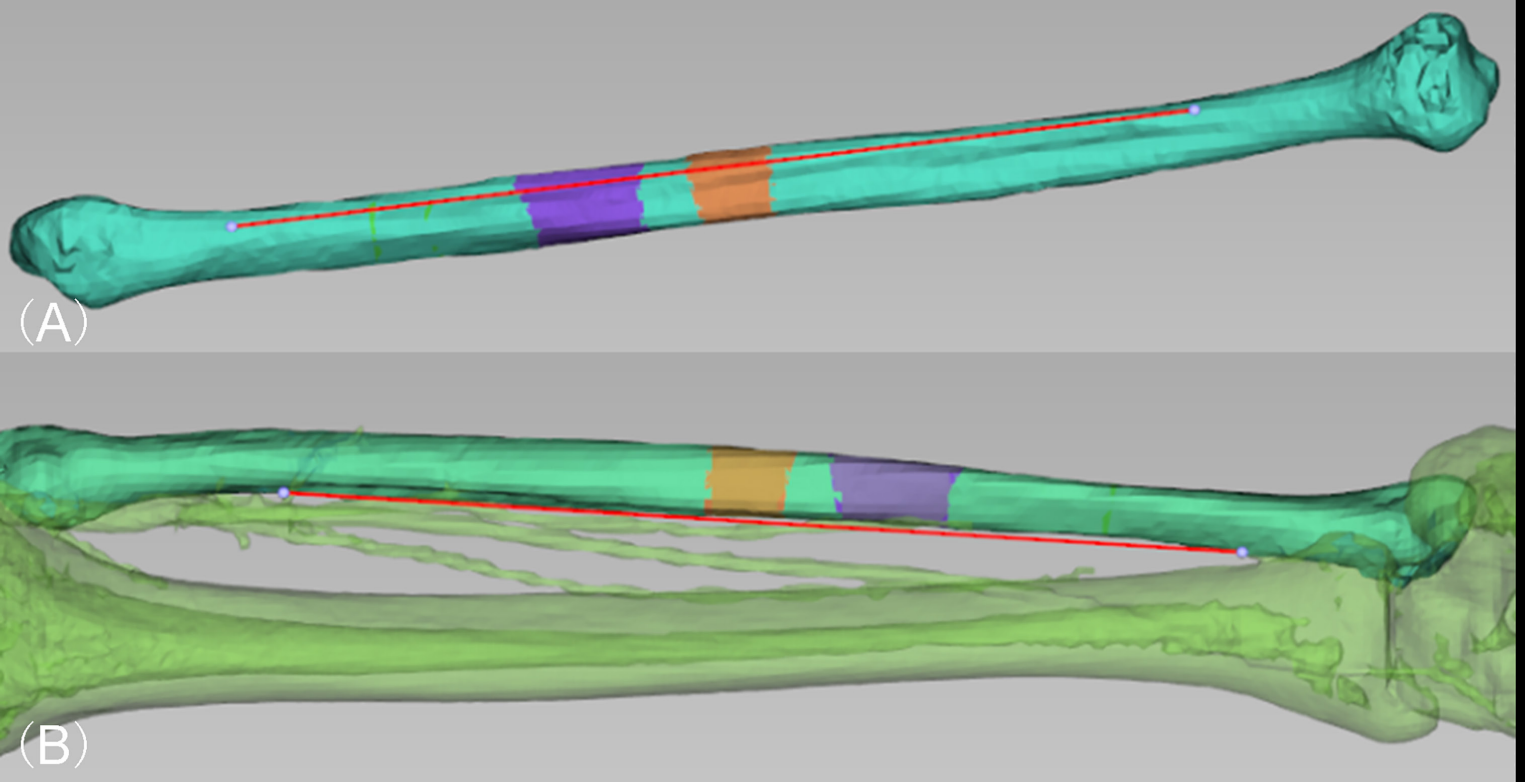
Virtual surgery simulation of designing the location fibular flap osteotomy. **(A)** Determination of proper osteotomy location and arrangement of all segments on the virtual fibula model (purple and orange). **(B)** The fibular flap was designed in consideration of preserving proper length at the distal end of the fibula as well as the location of proximal end of the peroneal artery.

Preoperative virtual surgical simulation and design of the CORPPP guiding template were conducted as previously described (13). First, imaging data were obtained from the patients’ maxillofacial region by large-field cone-beam computed tomography (CBCT; 0.25-mm slice interval; Imaging Sciences International 17-19 System, KaVo, USA) at intercuspal position, and from the fibular donor sites by Spiral CT (0.5-mm slice interval; SOMATOM Definition AS, SIEMENS, Germany). Then imaging data were loaded into the E3D CMF software (Digital Medicine and Virtual Reality Research Center, Central South University, China) for surgical simulations. Based on the mandibular lesion, the resection range was determined (**Figure 1A**), and simulated fibula segments were arranged into mandibular defects to fit the curved contour of the mandible (**Figure 1B**), generating the expected reconstructed mandibular model (**Figure 1C**). The fibular osteotomy was designed accordingly with consideration of proper length of the distal end of the fibula as well as the peroneal artery (**Figures 2A, B**).

**Figure 3:**
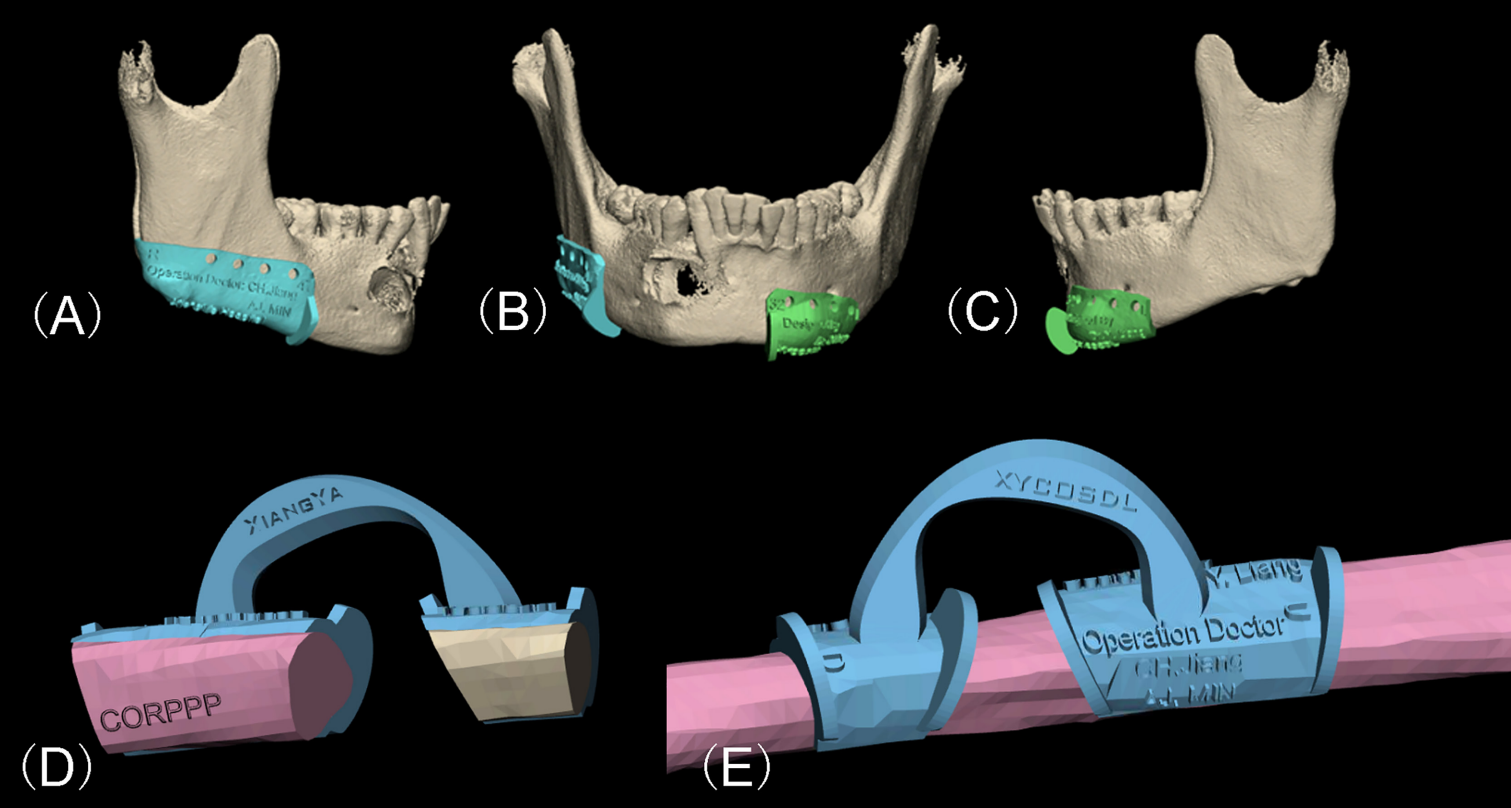
Virtual design of the CORPPP guiding template. **(A–C)** Lateral and frontal view of placement of CORPPP mandibulectomy guiding templates with guiding wings and designed fixing holes. **(D, E)** Medial and external view of the placement of CORPPP fibular osteotomy templates.

The titanium template was shaped and fixed to the ideal position with plastic ligatures on a 3D printout of the expected reconstructed mandibular model, and proper fixing holes were chosen from the titanium template. Then, the model was scanned again together with the titanium template by CBCT to generate the composite model. By overlapping the composite model and the expected model in the software, location of the fixing holes can simply be transferred onto both the remaining mandible as well as each fibula segment in expected model. Resection guiding templates (**Figures 3A** **–C**) and fibular osteotomy templates (**Figures 3D, E**) were then designed to include the designed fixing holes, and the guide wings (or grooves) of the templates were consistent with the expected osteotomy template. In this way, the fixing holes were unified, which play the dual role of fixing the guiding template to the bone surface and determine the position for the subsequent fixation of the titanium template.

**Figure 4:**
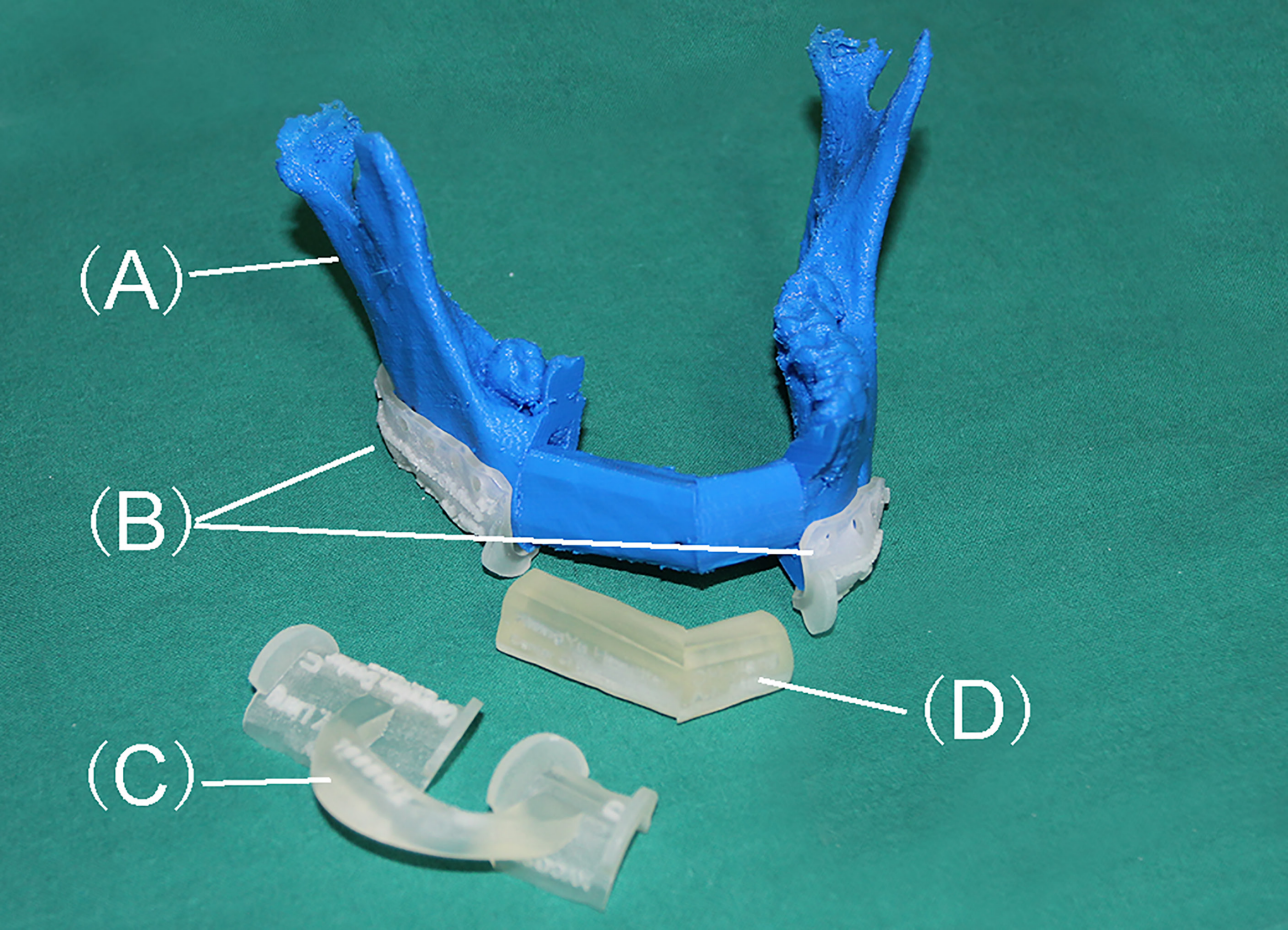
3D printout templates of models and CORPPP guiding templates. **(A)** Expected mandible model after reconstruction. **(B)** CORPPP mandibulectomy guiding templates with fixing holes. **(C)** CORPPP fibular osteotomy template. **(D)** CORPPP positioning template.

All the designed guiding templates are stored in Stereolithography (STL) documents and the 3D printout templates and models were fabricated using polymer nylon materials by Hunan Huaxiang Incremental Manufacturing Co. Ltd (**Figure 4**). The templates and the pre-shaped titanium template were then sterilized and prepared for further use.

### 2.3 Surgical Procedures and Postoperative Data Collection

**Figure 5:**
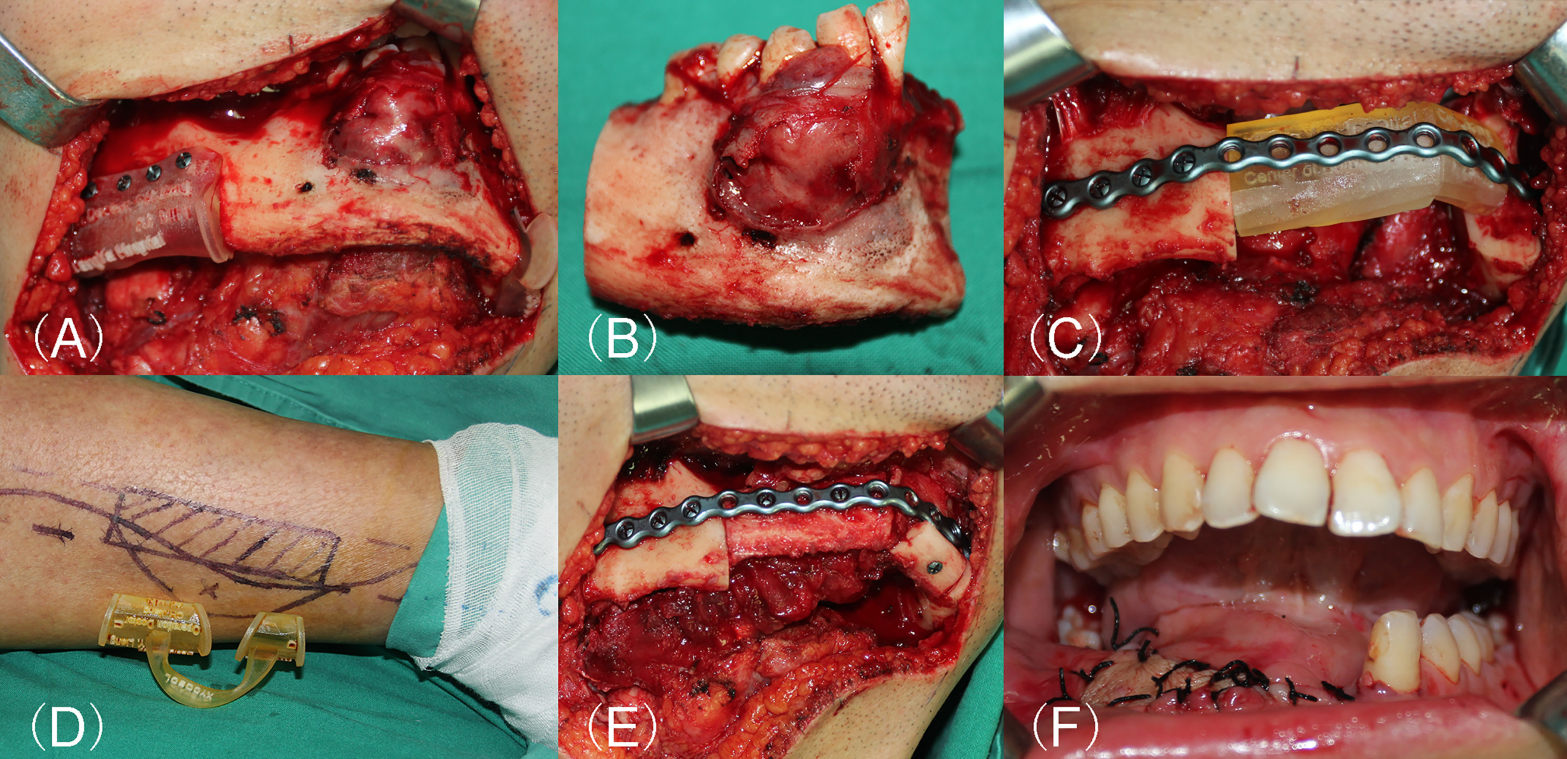
Surgical procedure of CORPPP-guided mandibular reconstruction. **(A)** Placement of the resection guiding templates with the designed fixing holes and screws. **(B)** Mandibular lesion specimen after segmental resection. **(C)** Placement of the pre-shaped titanium plate with the designed fixing holes. **(D)** Design of the fibular flap. **(E)** Fixation of fibula segments into the defect.

The mandibular reconstruction with fibular flaps were conducted as previously described (6, 13). For the patients in the CORPPP-guided group, segmental mandibulectomy was performed with the assistance of the resection guiding template (**Figures 5A, B**). Through the fixing holes, the mandible was drilled, and the fixing holes were used for fixation of the titanium plate to restore the correct occlusal relation and the position of the condyles (**Figure 5C**). At the same time, fibular flaps were harvested with the osteotomy template (**Figure 5D**). With the assistance of the positioning templates, the fibula segments were arranged and fixed as designed (**Figure 5E**). For patients in the freehand group, conventional procedures were performed without guiding templates. Vascular anastomosis was then processed, and the skin paddle was used to repair the defect as well as monitoring the circulation of the flap if needed (**Figure 5F**).

All patients received conventional free flap post-operative care and medication. During the routine follow-up visit at the 6th month after operation, CBCT scan was performed to obtain the post-operative data for deviation evaluation.

### 2.4 Virtual Model Superimposing and Deviation Evaluation

Post-operative image data were used to reconstruct the actual 3D model in the software. Superimposing of the actual model and the expected model was conducted using the model superimpose tracing function in 3D analysis module of the InVivoDental software (Version 5.2.4, Anatomage, USA).

#### 2.4.1 Deviation Measurement in CORPPP-Guided Mandibular Lesion Resection

To measure the deviation during mandibulectomy, both (1) the deviation of osteotomy angle and (2) the deviation of lesion resection line were measured. Three points were selected on the expected mandibular model in the software to form the designed osteotomy plane, and the actual osteotomy plane was formed on the post-operative model with the same method. The acute angle formed by the two planes was defined as the resection angle deviation. The inferior mandibular margin points were marked in both the actual model and the expected model, and the distance between them was defined as the deviation of the lesion resection line.

#### 2.4.2 Deviation Measurement in CORPPP-Guided Fibular Osteotomy

The length of all fibula segments was measured in both the expected mandibular model and the actual model in CORPPP-guided patients. The absolute value of the actual length minus the designed length was recorded as the deviations in CORPPP-guided fibular osteotomy.

#### 2.4.3 Overall Deviation Measurement of the Mandibular Anatomical Landmarks

Patients were divided into two groups based on whether the ramus was reconstructed. To evaluate the displacement and rotation of the remaining mandible, the maxillary was used for superimposing the pre- and postoperative virtual models. Mandibular anatomical landmarks including the condylar head point (Co.), gonion point (Go.), and coracoid process point (Cor.) on the ipsilateral side were chosen for measurement. Those points were marked on both the postoperative actual model and the preoperative expected model, and the distance was recorded as the overall deviation.

#### 2.4.4 Establishment of Coordinate System and Triaxial Deviation Measurement of the Mandibular Anatomical Landmarks

We applied coordinate conversion to analyze the deviations between the designed model and the actual model in triaxial directions. A coordinate system was established as follows. Plane A, an imaginary plane that was parallel to the Frankfort plane, was established at the level of the mandibular incisal notch. The midpoint of the line segment connecting the central fossa points of the bilateral mandibular first molars was projected to Plane A, forming the origin of the coordinate axis. In the first molar missing situation, the bilateral central fossa points were substituted in the order of bilateral outermost points of the condyle, bilateral points of the mandibular angle, or bilateral points of the sigmoid notch. The *x*-axis was defined as the horizontal axis passing through the origin and Plane A, and the positive direction was defined from the contralateral side to the ipsilateral side, and from right to left in patients with bilateral lesions by default. The *y*-axis was formed by connecting the origin and the aforementioned midpoint, and the positive direction was defined posteriorly. The *z*-axis was formed vertical to Plane A, defining the positive direction superiorly.

We further analyzed the overall deviations of each mandibular anatomical landmarks in three-dimensional directions. Matrix Laboratory (R2014B version, MathWorks, USA) was used to complete the coordinate conversion of the landmarks from the actual model and the expected model into the same coordinate system. Vectors of each point (from the actual model towards the expected model) were calculated respectively, generating the triaxial deviation, and the signs (positive or negative) of the coordinates represent the direction.

### 2.5 Statistical Analyses

Deviations were measured and presented as mean ± SD. Homogeneity of variance was evaluated by Levene Variance Equality Test first, and *t* test or Mann–Whitney test was performed accordingly. Statistical significance was reached for *p* < 0.05. All statistical analyses were performed using SPSS Version 19.0.

## 3 Results

All patients in both the CORPPP group and the freehand group underwent successful mandibular reconstruction. All flaps survived and postoperative recovery was uneventful. The design and preparation of the CORPPP guiding templates for all 13 cases were successful. In the CORPPP group, the lesions were resected in accordance with the preoperative designs, the pre-shaped titanium plates and titanium screws were placed smoothly, and the occlusal relation was well recovered. All patients came for follow-up visits as expected.

Deviations were inevitable despite the assistance of the guiding templates. The deviation of the resection line was 1.23 ± 0.98 mm with a maximum of 2.54 mm, while the deviation of the mandibular resection angle was 4.11° ± 2.60° and the maximum was 9.53°. The actual length of fibula segments was longer than the designed length in 7 cases (mean: 0.35 ± 0.32 mm, maximum: 1.12 mm), and shorter in 22 cases (mean: 1.53 ± 1.19 mm, maximum: 4.61 mm).

The overall deviation of the mandibular anatomical landmarks in cases without ramus reconstruction was measured and presented in **Table 2**. The deviations of all anatomical landmarks (Co., Go., and Cor.) in the CORPPP group (1.73 ± 1.13 mm, 1.86 ± 0.96 mm, and 2.54 ± 0.50 mm, respectively) were significantly smaller than that in the freehand group (6.71 ± 3.42 mm, 5.38 ± 1.71 mm, and 11.05 ± 3.24 mm, respectively). Similarly, the overall deviations of Co. and Go. in cases with ramus reconstruction were also compared. In the CORPPP group, among seven cases, the deviation of Co. was 3.57 ± 1.62 mm, and the deviation of Go. was 4.36 ± 1.68 mm. In the freehand group, the deviation of condylar head was 9.79 ± 4.74 mm, and the deviation of gonion was 15.17 ± 6.53 mm in eight cases (**Table 3**).

**Table 2:** Deviation comparison between CORPPP group and freehand group in patients without ramus reconstruction.

Group	Number of cases	Deviation of Co. (mm)	Deviation of Go. (mm)	Deviation of Cor. (mm)
Freehand	7	6.71 ± 3.42	5.38 ± 1.71	11.05 ± 3.24
CORPPP	6	1.73 ± 1.13*	1.86 ± 0.96*	2.54 ± 0.50*

*p < 0.05.

**Table 3:** Deviation comparison between CORPPP group and freehand group in patients with ramus reconstruction.

Group	Number of cases	Deviation of Co. (mm)	Deviation of Go. (mm)
Freehand	8	9.79 ± 4.74	15.17 ± 6.53
CORPPP	7	3.57 ± 1.62*	4.36 ± 1.68*

*p < 0.05.

The triaxial deviations of all mandibular anatomical landmarks of the ipsilateral side of the mandible in the CORPPP-guided patients were measured. Patients without ramus reconstruction (**Table 4**) and with ramus reconstruction (**Table 5**) were calculated separately. However, statistical analysis could not perform due to the limited case numbers.

**Table 4:** Triaxial deviation of mandibular anatomical landmarks in CORPPP-guided patients without ramus reconstruction.

Landmark	Triaxial deviation	Positive direction (mm)	Number of cases	Negative direction (mm)	Number of cases
Co.	*x*	0.76 ± 0.57	5	−0.35	1
	*y*	1.39 ± 1.47	4	−1.28 ± 1.50	2
	*z*	0.20 ± 0.18	4	−0.19 ± 0.11	2
Go.	*x*	0.77 ± 0.53	5	−0.18	1
	*y*	0.11 ± 0.14	2	−0.91 ± 1.04	4
	*z*	0.59 ± 0.49	5	−0.16	1
Cor.	*x*	1.67 ± 1.21	6	/	0
	*y*	0.86 ± 0.45	6	/	0
	*z*	1.27 ± 0.99	5	−0.52	1

**Table 5:** Triaxial deviation of mandibular anatomical landmarks in CORPPP-guided patients with ramus reconstruction.

Landmark	Triaxial deviation	Positive direction (mm)	Number of cases	Negative direction (mm)	Number of cases
Co.	*x*	1.72 ± 1.48	4	2.26 ± 2.15	3
	*y*	0.83 ± 0.63	4	1.21 ± 0.94	3
	*z*	1.11 ± 0.55	4	3.17 ± 3.00	3
Go.	*x*	2.78 ± 1.47	3	2.27 ± 2.14	4
	*y*	1.77 ± 0.95	3	1.30 ± 1.22	4
	*z*	2.60 ± 0.50	4	−0.69 ± 0.49	3

## 4 Discussion

### 4.1 Advantages and Limitations of the Template-Guided Mandibular Reconstruction

Digital surgical technology has yielded excellent clinical results in personalized functional mandibular reconstruction. Although surgical navigation is currently considered one of the best solutions for real-time confirmation in mandibular reconstruction, it can hardly be widely promoted due to the expensive equipment and time-consuming matching procedure (15).

Virtual surgery allows simulation of all the critical steps before the actual operation, including determination of the range for lesion resection, design of the fibula osteotomy, and the positioning and contouring of the fibular segments. By combining with the 3D printing technology, models and surgical guiding templates are designed and fabricated. In addition, it facilitates young surgeon training, and enhances the predictability and streamlining of surgery (7, 16, 17).

CORPPP technique, as previously designed and applied by our group, were specially designed for better positioning of the titanium plate and the seating of the bone grafts (13). The titanium plates were pre-shaped according to the 3D-printed mandible model preoperatively. Templates referring to each specific step are “printed-out” in advance, and the operation was processed step by step under the guidance. This advantage was also shared by other similar 3D-printed patient-specific surgeries. Gupta et al. reported that the total operation time was significantly reduced by nearly 1/3 in 3D-guided surgeries, which was 83.9 min in the 3D group and 124 min in the freehand group (18). Another study reported by Weitz et al. showed that the operations were 34 min shorter in virtually planned cases with optimizing accuracy (19). With the help of the models and templates, the intraoperative time was significantly reduced, improving the efficiency of the surgery and contributing to rapid recovery of patients.

The cost of the guiding template was mainly dependent on the type and the dosage of the material for 3D printing. Compared to other 3D-printed guiding templates, CORPPP neither changed the type nor increased the dosage of the material, and the manufacturing cost was generally the same as other guiding templates. In addition, the special fixing holes of CORPPP were designed to stabilize the guiding template to the bone surface as well as determine the position for the subsequent fixation of the titanium template. This special design can also be applied in combination with other similar 3D-printed guiding templates to reduce the skewing of the titanium plates, improve accuracy, and facilitate the reconstruction process. However, even under the guidance of the CORPPP guiding template, there were still inevitable deviations when each step was performed.

### 4.2 Deviation Analyses in CORPPP-Guided Mandibular Lesion Resection

By comparing the postoperative measurement with the preoperative virtual design, the average deviation of mandibulectomy line was 1.23 ± 0.98 mm, and the average deviation of osteotomy angle was 4.11° ± 2.60°. We believed that these deviations were produced by a tiny shift of the templates and the saw blades. The setting of the resection guiding template was designed by matching its inner surface with the specific structures on the external surface of the mandible. However, the chosen surface structures on the mandible were not so prominent that a tiny slippage of the template happens during positioning, which would lead to deviation of the mandibulectomy line. The simple solution was to increase the contact area between the template and the mandible surface, but expansion of the template would increase the stripping range of the remaining healthy mandible, resulting in loss of muscle attachment and periosteal blood supply, which might aggravate the mandibular ischemia, especially in patients with osteoradionecrosis.

As for the deviation of mandibulectomy angle, when performing mandibular resection, it was important to ensure that the saw blade travels along the guiding wings of the template to ensure accurate angulation of the osteotomy plane. However, during actual operation, the saw blade might tilt away from the wings when working in the blind field of the vision such as the lingual side of the mandible, thus causing a certain rotation of the mandibular osteotomy plane. Increased restriction and directional guidance of the saw blade path to ensure that the blade did not drift during osteotomy would reduce the deviation.

Integrating the above solutions, another template with guiding wing was added on the lesion side of the mandible, covering the mandibular lesion, and changing the design of the unilateral guiding wing to a guiding groove (17, 20). The contact area between the template and the mandible surface was greatly increased without extra stripping of the muscle attachment and periosteum. At the same time, the slot generated a stronger guiding effect on the saw blade, which effectively reduced the osteotomy angle deviation. Therefore, in our later recruited CORPPP cases, guiding grooves were used instead of wings, as shown in **Supplementary Figure 1**.

### 4.3 Deviation Analyses in CORPPP-Guided Fibular Osteotomy

In total, 28 fibular segments were measured, and the average length deviation was only 1.24 ± 1.17 mm, confirming the reliability and repeatability of CORPPP techniques. Interestingly, we found that most (22/28, 78.6%) of the fibular segments were shorter than the original design. The main reason was considered to be the excessive trimming during the fibula positioning. Similar to the mandibular resection step, deviations happened during fibular osteotomy, resulting in increased fibular length.

However, the positioning templates and the pre-shaped titanium plate were made strictly according to the design. Thus, the extra intercepted part of the fibula formed early contact points, resulting in changes in the overall length and the angles of the fibular segments, and the fixing holes on the fibula could not be matched with the corresponding holes on the pre-shaped titanium plate, so repeated trimming and grinding was frequently required. The fibula was stiff in texture and hard for trimming after osteotomizing into small segments. Ultimately, the grafted fibula segment would be shorter than designed. That was also the reason for the gaps between fibular segments found on review of the postoperative CBCT images.

### 4.4 Overall and Triaxial Deviation Analyses of Mandibular Anatomical Landmarks in CORPPP-Guided Mandibular Reconstruction

Conventional absolute deviations were measured by superimposing the reconstructed mandibles onto the preoperative virtual models based on the healthy side of the mandible. However, in the physiological state, the reconstructed mandible was distracted by muscles and adapted to occlusion, leading to physiological deviations on both the lesion side and the healthy side. Yang et al. discussed the novel algorithm for physiological deviation assessment. In the 3D-printed plate group, greater impact of temporomandibular joint (TMJ) deviations were found on the lesion side than the healthy side. Moreover, patients without preservation of both the condyle and ramus had significantly higher deviations of the condyle and joint space (21). This result was consistent with our study. We evaluated the physiological deviation on the ipsilateral side, and the CORPPP group showed significantly lower deviations of the mandibular anatomical landmarks on the ipsilateral side than the freehand group, suggesting that CORPPP technique improved the physiological position of reconstructed mandible.

The recruited 13 patients were divided into two groups according to whether the ramus was restored. There were two main reasons for grouping. Firstly, for measurement reason, due to the great difference in shape between mandible and fibula, anatomical landmarks on the residual mandible should be priorly selected for measurement to reduce the selection error, so the presence or absence of the ramus on ipsilateral side might affect the deviation (22, 23). Secondly, the deviations of the reconstructed mandible were assumed to be affected by the resection range, especially the posterior part of the mandible (24). The anatomical anchor of the fossa-condyle structure and the intact joint capsule stabilized the TMJ, and the masticatory muscles were mainly attached to the posterior mandible (21). Therefore, when the ramus was involved in the defect, the stability from the TMJ and the muscles greatly decreased, and reconstruction procedures became more complex at the same time, leading to increasing deviation.

It was generally accepted that the overall deviation was directly related to the final reconstruction outcome. Our result showed that among all the patients, no matter with or without ramus reconstruction, there were significantly smaller overall deviations of Co., Go., and Cor. in the CORPPP-guided group than the conventional free-hand group. Several studies have also reported less overall deviation of length and angles in the computer-aided group *versus* the conventional freehand group, when superimposing the virtual planning on post-operative mandible. Foley et al. reported the average deviation for free fibula flap 0.9 mm in the A–P dimension, 2.7 mm from condyle to condyle, and 2.5 mm from gonial angle to gonial angle in the transverse dimension (25). In a retropective study conducted by Zhang et al., the deviation in fibula segment length was 1.34 ± 1.09 mm, the angular deviation was 2.29° ± 1.19°, and the mean 3-D deviation was 0.53 ± 0.06 mm in the computer-aided group, indicating high accuracy in templated guided fibular flap mandibular reconstruction (26). Those findings were consistent and comparable with our results. As for esthetic evaluation, Bartier et al., in a CT symmetry study of 25 patients in the 3D group and 8 in the freehand group, found that deviations in the 3D group of the coronal mandibular angle, mandibular body height, and ramus length on the affected side were significantly lower, and the sagittal mandibular angle symmetry was better, indicating that 3D-guided mandibular reconstruction with fibula free flaps helped to restore a greater symmetry and improved esthetic outcome (27).

To find other potential causes for the offset of CORPPP-guided mandibular reconstruction, we further analyze the deviations of each mandibular anatomical landmarks in three-dimensional directions. Our results revealed that in almost all the CORPPP-guided cases with preserved ramus, deviations of the Cor. happened towards the medial, superior, and posterior directions, with the most pronounced displacement in the medial direction, averaging 1.26 mm. Unfortunately, the results of Co. and Go. could not be statistically analyzed due to the limited cases.

The reasons for the deviation of Cor. in CORPPP-guided cases were considered as follows. Firstly, intraoperative stripping of the muscles attached to the surface of the mandible affected the force balance of masticatory muscle groups. Therefore, under the traction of the temporalis muscle, posterior–superior displacement of the coracoid process happened. Secondly, after detailed observation of the overlapping images in the InVivoDental software, it was found that the inward migration was not only present at the coracoid process, but the entire anterior edge of the ramus had the tendency to rotate inward in some cases. The inward rotation also led to an inadequate fit of the ramus to the titanium plate, indicating that the ramus was not sufficiently stable during fixation.

The commercialized mandibular reconstruction plate that is commonly used consisted of two parts: the horizontal part and the ascending part, which were connected by an arched turn of nearly 130°. Usually, the screws on the ascending part were arranged in a linear pattern after ramus fixation, which was not stable enough. In addition, the pre-shaped titanium plate did not fit perfectly to the surface of the mandible, so rotation might occur during fixation. Since the rotation towards the lateral side was blocked by the titanium plate, the anterior edge of the ramus could only rotate towards internally.

Therefore, when designing the fixation position of the titanium plate, we should consider appropriate posterior displacement and try to use a few nail holes on the horizontal part of the titanium plate to increase the stability of ramus fixation. It was also possible to increase the fitting of the titanium plate to the surface of the mandible and to reduce the forces exerted on the mandible by the titanium plate and screws in additional directions during fixation.

With further advances in 3D printing technology, personalized mandibular titanium plates can also be printed for better fitting, higher reconstruction accuracy (28). Based on preoperative CT-scan data, personalized titanium plate can be precisely preformed. Even for patients who underwent double-barrel fibular flap reconstruction, a special “one-piece” reconstruction plate has been reported to fix both barrels simultaneously, achieving satisfactory outcomes (29). After being sterilized, those personalized plates can be used directly without the need for bending. Moreover, it provides stronger guiding for the alignment and fixation of bone segments that may help reduce the deviations.

This study suggested that the CORPPP technique significantly improved the predictability, convenience, and accuracy of mandibular reconstruction. However, more clinical cases were required for further dimensional deviation analysis. The application and exploration of clinical practice would also continuously improve the design of templates.

## Data Availability Statement

The raw data supporting the conclusions of this article will be made available by the authors, without undue reservation.

## Ethics Statement

Written informed consent was obtained from the individual(s) for the publication of any potentially identifiable images or data included in this article.

## Author Contributions

JC: Writing—original draft preparation, writing—review and editing. RZ: Data curation, formal analysis, resources, and software. YL: Conceptualization, visualization, software, and methodology. YM: Writing—original draft and writing—review and editing. SS: Writing—original draft and writing—review and editing. CJ: Conceptualization, funding acquisition, surgical operator supervision, methodology, and validation. All authors contributed to the article and approved the submitted version.

## Conflict of Interest

The authors declare that the research was conducted in the absence of any commercial or financial relationships that could be construed as a potential conflict of interest

The reviewer W-FY declared a past co-authorship with one of the authors CJ to the handling editor.

## Publisher’s Note

All claims expressed in this article are solely those of the authors and do not necessarily represent those of their affiliated organizations, or those of the publisher, the editors and the reviewers. Any product that may be evaluated in this article, or claim that may be made by its manufacturer, is not guaranteed or endorsed by the publisher.
