# Supplementary figures and images for: Deviation Analyses of Computer-Assisted, Template-Guided Mandibular Reconstruction With Combined Osteotomy and Reconstruction Pre-Shaped Plate Position Technology: A Comparative Study
Source: Front Oncol. 2021 Oct 27;11:719466. doi: 10.3389/fonc.2021.719466 (PMC8579124; doi:10.3389/fonc.2021.719466)

## Slide 1
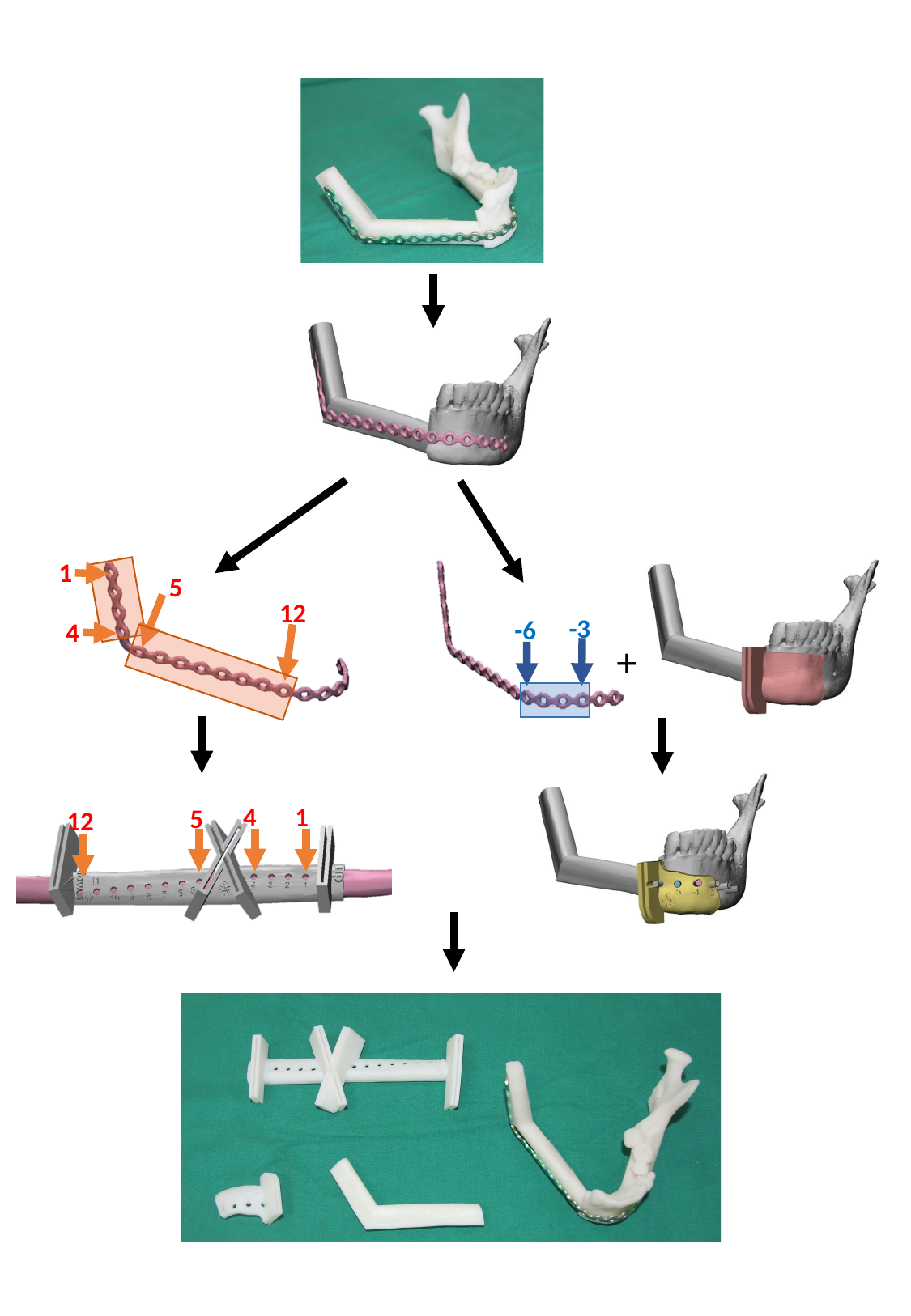

-3
-6
1
5
12
4
+
4
1
5
12

Supplement: Supplementary file 1 [file Presentation_1.pptx]
